# New species and new records of earthworms of the genus *Drawida* from Kerala part of the Western Ghats biodiversity hotspot, India (Oligochaeta, Moniligastridae)

**DOI:** 10.3897/zookeys.691.13174

**Published:** 2017-08-17

**Authors:** S. Prasanth Narayanan, S. Sathrumithra, G. Christopher, J.M. Julka

**Affiliations:** 1 Advanced Centre of Environmental Studies and Sustainable Development, Mahatma Gandhi University, Priyadarsini Hills, Kottayam – 686560, Kerala, India; 2 School of Biological and Environmental Sciences, Faculty of Basic Sciences, Shoolini University, Solan – 173212, Himachal Pradesh, India

**Keywords:** Annelida, Clitellata, description, distribution, habitat, soil fauna, taxonomy

## Abstract

Two new species of *Drawida* Michaelsen, 1900, namely *Drawida polydiverticulata* Narayanan & Julka, **sp. n.** and *Drawida thomasi* Narayanan & Julka, **sp. n.**, are described from material collected from the Indian state of Kerala, which lies in the Western Ghats biodiversity hotspot. *Drawida elegans* Rao, 1921, *Drawida kanarensis* Stephenson, 1917, *Drawida modesta* Rao, 1921, *Drawida somavarpatana* Rao, 1921, and *Drawida thurstoni* Gates, 1945 are recorded for the first time from the state.

## Introduction

Kerala is a narrow coastal equatorial tract of India (between 8°17'–12°47'N and 74°52'–77°24'E). The steep sloping land of Kerala along the southwest corner of the Indian Peninsula has its own unique identity (Nair 2011). The dominating surface feature of the state is the Western Ghats, which is one of the eight ‘hottest hotspots’ of biodiversity in the world (Myers et al. 2000, Mittermeier et al. 2011). In a broad generalized approach, Kerala can be divided into three distinct physiographic regions, namely the coastal lowlands (< 75 m a.s.l.), midlands (75–500 m a.s.l.), and high ranges (500–2000 m a.s.l.) (Iype et al. 1991). The area experiences two rainy seasons, viz., the southwest monsoon (June to September) and northeast monsoon (October to November). Annual rainfall ranges from 1,520 to 4,075 mm, but it may be as high as 6,000 mm in certain pockets and as low as 600 mm in rain shadow areas (KSCSTE 2007). The general climate is mostly tropical but tends to be temperate in high mountainous areas (average temperature 19°C–37°C; minimum 0°C in high ranges). Major forest types are tropical evergreen and semi evergreen, tropical moist and dry deciduous, mountain ‘sholas’, grasslands and low land scrub jungles (Islam and Rahmani 2004).

A great variety of vegetation coupled with high rainfall and moderate temperature has created a cradle for earthworm diversity in Kerala, which harbours about 21% of country’s known earthworm species (Narayanan et al. 2016). It is noteworthy that Kerala also possesses about 40% of earthworm species found in the Western Ghats that constitute India’s mega earthworm diversity area with 200 species (Julka and Paliwal 2005, Julka et al. 2009, Narayanan et al. 2016) of the 505 species from the Indian region (Blakemore 2007).

Several species in Kerala are known only from the original description, and most of them were recorded more than 80–90 years ago (Narayanan et al. 2016). Hence, we conducted extensive survey of earthworms in diverse habitats in the coastal areas, midlands and various types of forests in the hilly regions of the state. This has revealed the presence of two new species and five new records of the genus *Drawida* Michaelsen, 1900. The details of the two new species *Drawida polydiverticulata* sp. n. and *Drawida thomasi* sp. n. and the newly recorded *Drawida elegans* Rao, 1921, *Drawida kanarensis* Stephenson, 1917, *Drawida modesta* Rao, 1921, *Drawida somavarpatana* Rao, 1921, and *Drawida thurstoni* Gates, 1945 are dealt with in this paper.

## Materials and methods

Earthworms were obtained from soil by digging and hand sorting methods, and also searching organic microhabitats such as fallen tree trunks and leaf litter. Specimens were fixed in 5% formalin and subsequently transferred to ethanol. All anatomical observations were made by dissection under a stereomicroscope (Nikon SMZ800N), and illustrations were made by the attached drawing tube. Holotype and paratype specimens of the new species have been deposited at Zoological Survey of India, Western Ghats Regional Centre (ZSI-WGRC), Kozhikode (Calicut), Kerala, India. Other specimens are housed in Advanced Centre of Environmental Studies and Sustainable Development (ACESSD), Mahatma Gandhi University, Kottayam, Kerala, India.

## Systematic studies

### Genus Drawida

Taxon classificationAnimaliaOligochaetaMoniligastridae

Michaelsen, 1900

#### Type species.

*Drawida barwelli* Michaelsen, 1900

### Drawida polydiverticulata

Taxon classificationAnimaliaOligochaetaMoniligastridae

Narayanan & Julka sp. n.

http://zoobank.org/90E6A8F1-6BBD-4598-A93B-777BAF638391

#### Type material.

*Holotype*. Clitellate (Reg. no. ZSI/WGRC/IR/INV-8835), Meenthottychola (10°10'21.4"N; 77°02'2.3"E) in Eravikulam National Park, Idukki District, Kerala State, India, 2010 m a.s.l., stream side in shola forest, 22 November 2016, S.P. Narayanan, S. Sathrumithra and G. Christopher coll.

*Paratype*. 6 clitellate (Reg. no. ZSI/WGRC/IR/INV-8836); same collection data as for holotype.

#### Other material.

Two aclitellate (ACESSD/EW/721), Pullaradichola (10°11'33.4"N; 77°12'9.7"E) in Anamudi Shola National Park, Idukki District, Kerala State, India, 2113 m a.s.l., from the side of a water logged area within grassland, where recently the exotic wattle plantations has been clear felled, 25 May 2013, S.P. Narayanan, T. Augustine, A. Sasi and S. Sathrumithra coll.; 3 aclitellate (ACESSD/EW/722), Mattuchola (10°14'28.7"N; 77°14'12.9"E) in Chinnar Wildlife Sanctuary, Idukki District, Kerala State, India, 1954 m a.s.l., stream side in a grassland, 24 November 2013, T. Augustine, D. Kuriakose, S. Sathrumithra and S.P. Narayanan coll.; 4 aclitellate (ACESSD/EW/723), Pettymudy forest camp shed area (10°10'26.7"N; 77°01'25.6"E) in Eravikulam National Park, Idukki District, Kerala State, India, 1966 m a.s.l., stream side within shola forest, 21 November 2016, S.P. Narayanan, S. Sathrumithra and G. Christopher coll.

#### Diagnosis.

Length 50–73 mm, diameter 4–5 mm, segments 120–136. Colour bluish. Male pores in 10/11, at centres of oval porophores, at about mid bc. Spermathecal pores in 7/8 at c lines. Genital markings absent. Gizzards number 3–5 in 12–17. Coiled vas deferens mass about one fourth to half of testis sac; vas passing directly into prostate dorsally at about its middle. Prostates glandular ovate and erect, prostatic capsule club-shaped. Spermathecal atria erect in segment 7, each with 4–6 ental lobes. Nephridiopores aligned with d.

**Figure 1.**
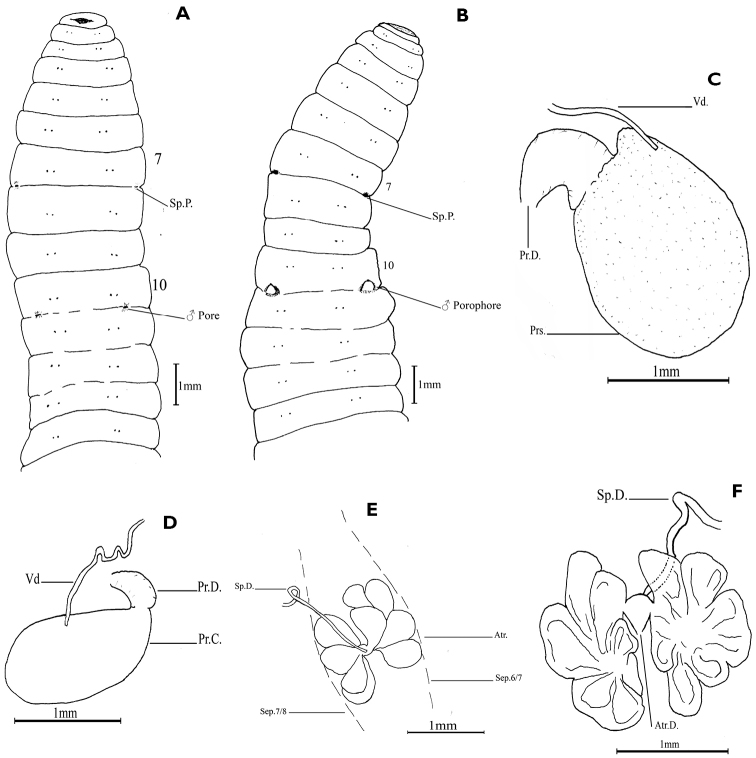
*Drawida polydiverticulata* sp. n. **A** Holotype - ventral view **B** Paratype – ventral view **C** Prostate – ventral view (gland uplifted) **D** Prostatic capsule – dorsal view **E** Spermathecal atria - dorsal view **F** Spermathecal atria - ventral view. Abbreviations: Atr. – Atrium; Atr.D. – Atrial duct; Pr.C. – Prostatic capsule; Prs. – Prostate; Pr.D. – Prostatic duct; Sep. – Septum; Sp.D. – Spermathecal duct; Sp.P. – Spermathecal pore; Vd – Vas deferens.

**Description**. Colour bluish (bluish pigmentation in circular muscle layer); body circular in cross section. Dimension: Holotype – 73 mm by 4 mm at segment 9, 120 segments; paratypes – 50–72 mm by 4–5 mm at segment 9, 125–136 segments. Setae lumbricine, closely paired, present from segment 2; setal formula aa = 8–15.2 ab = 1.05–1.95 bc = 13.33–19 cd = 0.28–0.45 dd at segment 8, aa = 11.5–12.67 ab = 0.92–1.08 bc = 9.2–9.5 cd = 0.26–0.31 dd at segment 20. Clitellum annular, at segments 10–13 (4 segments), intersegmental furrows distinct, colour reddish. Spermathecal pores paired, small transverse slits, with tumescent lips, at intersegmental furrow 7/8, aligned with setae c; males pores paired, minute, at intersegmental furrow 10/11, each pore at centre of oval porophore at about mid bc (Fig. 1A, B). Genital markings absent. Nephridiopores present from segment 3, aligned with setae d.

Septa 5/6/7/8/9 slightly muscular. Gizzards 4 in segments 12–15 (holotype), smallest in segment 12 increasing in size progressively; number variable in other specimens, 3 in segments 12–14 or 13–15 (Meenthottychola specimens), 3 in segments 13–15 or 4 in segments 12–15 (Pettymudy specimens), 4 in segments 13–16 (Pullaradichola specimens), 5 in segments 13–17 (Mattuchola specimens); intestine begins in segment 22±1. Last pair of hearts in segment 9; commissures of extra oesophageal vessels present on posterior face of septa 8/9 and 9/10. Testis sacs paired, mostly in segment 10, extending to segment 11; vas deferens long, coiled in a number of hairpin loops aggregated to form a mass about one fourth to half the size of testis sac, passing directly into prostate dorsally at about its middle. Prostates paired, glandular, ovate, erect (may be bent on itself anteriorly or posteriorly) (Fig. 1C); prostatic capsule smooth, club shaped (Fig. 1D); prostatic duct short, slender, narrowed before entering parietes. Spermathecae paired in segment 8; atrium erect in segment 7 and with 4–6 ental lobes arranged in two groups (Fig. 1E, F), spermathecal duct discharges at junction of common ducts of two groups. Ovarian chamber complete, ovisacs short, extending to segment 12–13. Nephridia holoic, avesiculate; functional at segment 10.

#### Etymology.

The specific epithet ‘*polydiverticulata*’ is after multi-lobed condition of spermathecal atrium.

#### Type locality.

Meenthottychola (10°10'21.4"N; 77°02'2.3"E) in Eravikulam National Park, Idukki District, Kerala State, India, 2010 m a.s.l., 23 km away from Munnar town, stream side in shola forest. Common vegetation of this region is dominated by *Syzygium arnottianum*, *Ilex denticulata*, *Michaelia nilagirica*, *Elaeocarpus recurvatus*, and *Microtropis ramiflora*.

#### Ingesta.

Coagulum comprising of mineralized soil, rootlets, tiny pieces of bark and leaves.

#### Distribution.

**Figure 2.**
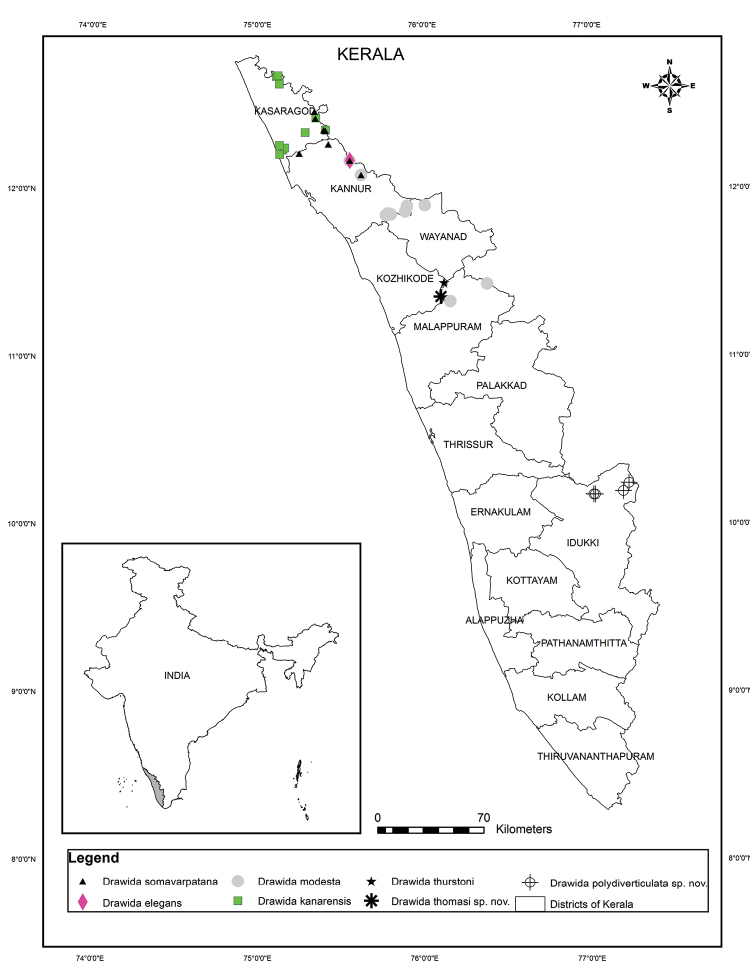
Distribution of newly described and reported *Drawida* species from Kerala.

India: Kerala: District Idukki: Meenthottychola and Pettymudy in Eravikulam National Park, Pullaradichola in Anamudi Shola National Park and Mattuchola in Chinnar Wildlife Sanctuary (Fig. 2).

#### Habitat.

Shola forest in vicinity of streams; near to stagnant pools or streams in grass lands.

#### Remarks.

*Drawida polydiverticulata* sp. n. is distinguishable from all the known species of the genus in having spermathecal atrium with more than two lobes. In one specimen from Pettymudy, spermatheca on right side with one branch of atrium along with lobes extends to segment 8.

### Drawida thomasi

Taxon classificationAnimaliaOligochaetaMoniligastridae

Narayanan & Julka sp. n.

http://zoobank.org/319B601A-CEDE-4AD6-89AA-56056C42FC44

#### Type material.

*Holotype*. Clitellate (Reg. no. ZSI/WGRC/IR/INV-8837), Kozhippara waterfalls (11°21'14.5"N; 76°6'29.2"E) near Kakkadampoyil, Malappuram District, Kerala State, India, 541 m a.s.l., by the side of tuber cultivated field earlier used for coffee plantation, 29 October 2014, S.P. Narayanan and S. Sathrumithra coll.

*Paratype*. One clitellate and one aclitellate (Reg. no. ZSI/WGRC/IR/INV-8838), same collection data as holotype.

#### Diagnosis.

Length 55–66 mm, diameter 4.5 mm, segments 66–105. Colour bluish. Male pores in 10/11, large transverse slits, slightly lateral to b lines. Spermathecal pores in 7/8 at c lines. Genital markings absent. Gizzards number 3 in 15–17. Coiled vas deferens mass as large as testis sac; vas discharging directly into prostate dorsally at about its middle. Prostates glandular tubular, slightly bent, prostatic capsule tubular, bent entally. Spermathecal atria bilobed, one lobe in segment 7 and the other in segment 8; atrial lobes tubular, very long, 9–10 mm in length, coiled into compact masses. Nephridiopores aligned with d.

#### Description.

**Figure 3.**
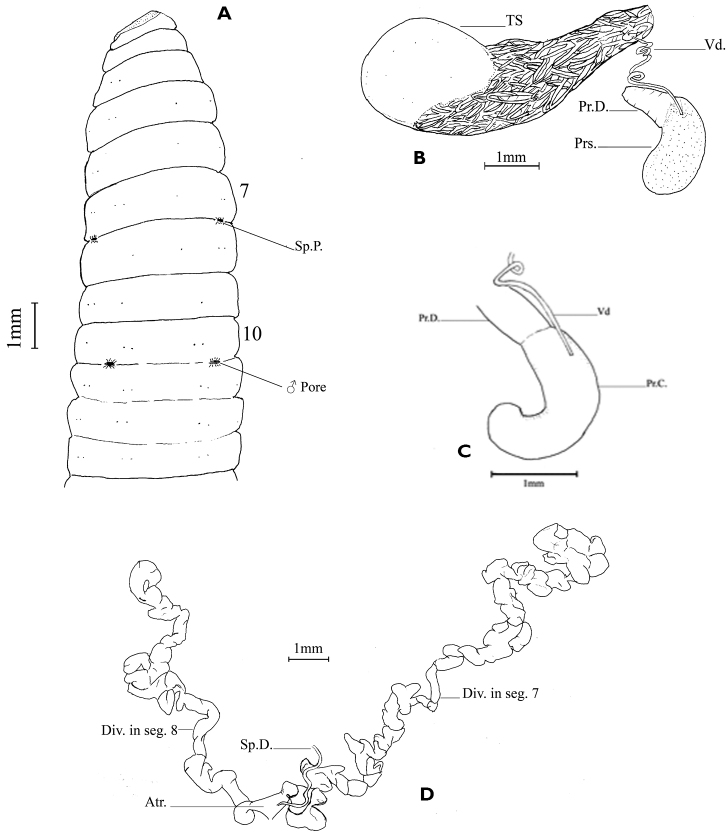
*Drawida thomasi* sp. n. **A** Holotype - ventral view **B** Prostate and testis sac **C** Prostatic capsule **D** Spermathecal atria - partially uncoiled. Abbreviations: Atr. – Atrium; Div. in seg. – diverticula in segment; Pr.C. – Prostatic capsule; Prs. – Prostate; Pr.D. – Prostatic duct; Sp.D. – Spermathecal duct; Sp.P. – Spermathecal pore; TS – Testis sac; Vd – Vas deferens.

Colour bluish; body circular, slightly flattened dorsoventrally. Dimension: Holotype 66 mm by 4.5 mm at segment 9, 105 segments; paratypes 55–57.5 mm by 4.5 mm at segment 9, 66–96 segments. Setae lumbricine; some setae on anterior segments may be absent (Table 1); setae ab enlarged on segment 8 and posterioriad segments (Fig. 3A); setal formula aa = 5–7.5 ab = 1.11–3 bc = 5.71–7.5 cd = 0.17–0.31 dd at segment 8, aa = 6.25–14.5 ab = 0.71–1.11 bc = 8.33–14.5 cd = 0.24–0.29 dd at segment 20.

**Table 1.** Presence (√) or absence (x) of setae on some anterior segments of the type materials.

Holotype	Setae on left side				Setae on right side			
Segment	d	c	b	a	a	b	c	d
2	x	x	x	x	x	x	x	√
3	x	x	x	x	x	x	x	√
4	x	x	x	x	√	√	x	√
5	√	x	x	√	x	√	√	√
6	√	√	x	√	x	x	√	√
7	√	x	x	√	√	√	x	x
8	√	√	x	√	√	x	√	√
9	√	√	√	x	x	√	x	√
10	√	√	√	√	√	√	√	√
**Paratype 1**	**Setae on left side**				**Setae on right side**			
Segment	d	c	b	a	a	b	c	d
2	√	x	x	x	x	x	√	√
3	√	√	x	√	√	x	√	X
4	√	√	√	√	x	x	√	√
5	√	√	√	√	√	√	x	√
6	√	√	√	√	√	√	√	√
7	√	√	√	√	x	x	√	√
8	√	√	√	√	√	√	√	√
9	√	√	x	√	√	√	√	√
10	√	x	√	√	√	√	x	x
**Paratype 2**	**setae on the left side**				**setae on the right side**			
Segment	d	c	b	a	a	b	c	d
2	√	x	x	x	x	x	x	x
3	√	x	x	√	x	√	x	√
4	√	x	√	x	x	x	√	√
5	x	x	x	x	√	x	x	x
6	x	x	√	√	√	x	x	x
7	x	√	x	√	x	√	x	√
8	√	x	√	√	x	√	√	√
9	√	√	x	x	x	√	√	√
10	√	√	x	x	√	√	√	√

Clitellum annular, at segments 10–13 (= 4 segments), indicated by reddish colour and slight swelling, intersegmental furrows distinct. Spermathecal pores paired, small transverse slits at setae at intersegmental furrow 7/8, aligned with setae c; male pores paired, large transverse slits at intersegmental furrow 10/11, slightly lateral to setae b lines. Genital markings absent. Nephridiopores, present from segment 3, aligned with setae d.

Septa 5/6/7/8/9 slightly muscular. Gizzards 3 in segments 15–17 (holotype and paratypes); intestine begins in segment 24. Last pair of hearts in segment 9; commissures of extra oesophageal vessel present on posterior face of septum 8/9, not recognizable on posterior face of 9/10. Testis sacs paired, in segments 9 and 10, extending to segment 15 on left side and to segment 17 on right side; vas deferens long, coiled in hairpin loops, aggregated into a mass as large as testis sac, discharging directly at about middle of dorsal face of prostate. Prostates paired, glandular, tubular, slightly bent at ental end (Fig. 3B); prostatic capsule shining, smooth, tubular, slightly bent entally (Fig. 3C); prostatic duct about half as long as gland, thick, slightly narrowed before entering parietes. Spermathecae paired, in segment 8; atrium bilobed, one lobe in segment 7 and the other in segment 8, each lobe tubular, very long and coiled into a compact mass occupying entire body cavity of respective segment, 9–10 mm long (when uncoiled); spermathecal duct short with a few coils entering at junction of two atrial lobes (Fig. 3D). Ovarian chamber incomplete; ovisacs paired extending back to segment 16. Nephridia holoic, avesiculate; functional at segment 10.

**Etymology**. Named after Prof. (Dr.) A.P. Thomas, who initiated taxonomic studies on the earthworms of Kerala state at Advanced Centre of Environmental Studies and Sustainable Development, Mahatma Gandhi University.

#### Type locality.

Kozhippara waterfalls (11°21'14.5"N; 76°6'29.2"E) near Kakkadampoyil, 27 km away from Nilambur town, Malappuram District, Kerala State, India, 541 m a.s.l., by the side of tuber-ultivated field earlier used for coffee plantation.

#### Distribution.

Known only from the type locality (Fig. 2).

#### Ingesta.

Mostly silt, with tiny pieces of mica and organic material.

#### Biology.

Autotomy is very common. Infested with nematodes in the region of reproductive system.

#### Habitat.

Bushes with grassy under growth, loamy soil, rich in organic matter, by the side of tuber cultivated field earlier used for coffee plantation.

#### Remarks.

*Drawida thomasi* sp. n. belongs to a group of species of *Drawida* with glandular prostates and bilobed spermathecal atria. It can be easily distinguished from other members of the group, *D. robusta robusta* (Bourne, 1887), *D. robusta ophidioides* (Bourne, 1894), *D. ghatensis* Michaelsen, 1910, and *D. somavarpatana* Rao, 1921 by the characteristics as given in Table 2.

**Table 2.** Comparison of *Drawida thomasi* sp. n. with related species.

Character	*D. robusta robusta* (Bourne, 1887)	*D. robusta ophidioides* (Bourne, 1894)	*D. ghatensis* Michaelsen, 1910	*D. somavarpatana* Rao, 1921	*D. thomasi* sp. n.
Length (mm)	136–200	310	80–160	85 (81–133^§^)	55–66
Diameter (mm)	6	7	2–6	4 (5–6^§^)	4.5
Segments	150–160	200	145–150	124 (104–154^§^)	66–105
Male pores	Nearer to c setal lines	Nearer to c setal lines	About midway between b and c setal lines	Nearer to b setal lines	Nearer to b setal lines
Spermathecal atrium	Distinctly bilobed; lobes erect, tubular; one lobe in segment 7 and the other in segment 8, anterior lobe larger	Distinctly bilobed; lobes teat like, one lobe in segment 7 and the other in segment 8, anterior lobe smaller	Slightly bilobed; lobes slight protuberances, confined to segment 7	Distinctly bilobed; lobes cylindrical, one lobe in segment 7 and the other in segment 8; both lobes of almost equal length	Distinctly bilobed; lobes tubular, one lobe in segment 7 and the other in segment 8; each lobe very long, coiled into a compact mass, occupying the entire body cavity of segment
Testis sacs	Confined to segments 9 &10	Confined to segments 9 &10	Extend to segments 13–14	Extend to segment 14	Extend to segments 15–17
Prostates	Hemispherical, sessile	Hemispherical, sessile	Thickly pear-shaped?	Bifid; lobes erect, finger like	Tubular, erect, slightly bent entally
Opening of Vas into prostate	Ental end of prostate^¶^	?	?	Ectal end of prostate^#^	About middle of prostate
Number of gizzards (segment location)	4 (12,13–16)	3 (14–16)	4 (16–19)	3 (16–18)	3 (15–17)

§ Based on the present study; ¶ As shown in the figure 5 of *Moniligaster indicus* Benham, 1893 in Bourne (1894), *Moniligaster indicus* is a synonym of *Drawida robusta robusta* (Michaelsen 1900); # As observed by one of the author (JMJ) during his studies on the types (syntypes) of *Drawida somavarpatana* (Reg. no. W416/1) in ‘National Zoological Collection’ at Zoological Survey of India, Kolkata.

### Newly recorded species of *Drawida* from Kerala state

#### Drawida elegans

Taxon classificationAnimaliaOligochaetaMoniligastridae

Rao, 1921

Drawida elegans Rao, 1921. Ann. Mag. Nat. Hist. (ser. 9), 8: 519.
Drawida elegans , Stephenson 1923. Fauna Br. India, Oligochaeta: 137.

##### Material examined.

7 aclitellate (ACESSD/EW/404), Paithalmala (12°10'1.7"N; 75°33'31.1"E), Kannur district, Kerala, India, 1076 m a.s.l., higher altitude evergreen forest, 30 November 2012, S.P. Narayanan, T. Augustine and S. Sathrumithra coll.

##### Diagnosis.

Length 130 mm, diameter 5 mm, 206 segments. Setae aa = 20 ab = 1.2 bc = 20 cd on segment 7, aa = 27 ab = 1.7 bc = 27 cd on mid body segments. Male pores paired, small, at 10/11, slightly lateral to setae b lines, at centres of slightly raised oval papillae. Spermathecal pores paired, at 7/8, at setae c lines. Gizzards 5, in segments 12–16. Prostates glandular, sessile, elongated; vas deferens long, coiled into a mass of hairpin loops, discharging directly at ental end of prostate. Spermathecae paired, in segment 8; atrium large, shortly pear-shaped, narrower ectal end; spermathecal duct discharging at ental end of atrium.

##### Distribution.

India: Kerala: District Kannur: Paithalmala (new record) (Fig. 2); Karnataka: District Kodagu (Coorg): Bhagamandla, Coorg Hills (Rao 1921).

##### Remarks.

Range of the length, diameter, and number of segments in the Kerala specimens are 83–117 mm, 5–6 mm, and 161–171 respectively. Three gizzards in the present specimens in segments 12–14. Mass of vas deferens loops is shorter than testis sac.

#### Drawida kanarensis

Taxon classificationAnimaliaOligochaetaMoniligastridae

Stephenson, 1917

Drawida kanarensis Stephenson, 1917. Rec. Indian Mus., 13: 364.
Drawida kanarensis , Stephenson 1923. Fauna Br. India, Oligochaeta: 143.

##### Material examined.

2 clitellate, 2 aclitellate (ACESSD/EW/170), Ranipuram (12°25'18.2"N; 75°21'14.4"E), Kasaragod district, Kerala, India, 935 m a.s.l, evergreen forest, 19 October 2012, S.P. Narayanan, S. Sathrumithra and M. Ramesan coll.; 1 aclitellate (ACESSD/EW/174), Periyathaduka - Padre (12°37'26.6"N; 75°7'58.4"E), Kasaragod district, India, 89 m a.s.l., Arecanut plantation, 18 October 2012, S. Sathrumithra, S.P. Narayanan and M. Ramesan coll.; 1 clitellate (ACESSD/EW/175), Adakasthala - Perla (12°40'9.6"N; 75°6'54.3"E), Kasaragod district, Kerala, India, 77 m a.s.l., cultivated area near to river, 18 October 2012, S.P. Narayanan, S. Sathrumithra and M. Ramesan coll.; 3 clitellate, 2 aclitellate (ACESSD/EW/176), Pandigaya - Perla (12°40'27"N; 75°07'27.8"E), Kasaragod district, Kerala, India, 102 m a.s.l., rubber plantation, 18 October 2012, S.P. Narayanan, S. Sathrumithra and M. Ramesan coll.; 4 clitellate, 4 aclitellate (ACESSD/EW/177), Kottencheri (12°20'57.6"N; 75°24'45.9"E), Kasaragod district., India, 801 m a.s.l., evergreen forest, 20 October 2012, S.P. Narayanan, T. Augustine, S. Sathrumithra and M. Ramesan coll.; 3 clitellate, 3 aclitellate (ACESSD/EW/178), Plachikkara (12°20'6.2"N; 75°17'19.8"E), Kasaragod district, Kerala, India, 56 m a.s.l., degraded forest, 20 October 2012, T. Augustine, S.P. Narayanan and M. Ramesan coll.; 1 clitellate (ACESSD/EW/179), Veeramalakunnu hillock (12°13'49.8"N; 75°9'15.8"E), Kasaragod district, Kerala, India, 13 m a.s.l., dense scrub land with exotic *Acacia auriculiformis* trees, 21 October 2012, S.P. Narayanan, M. Ramesan, T. Augustine and S. Sathrumithra coll.; 5 clitellate (ACESSD/EW/180), Melerippukavu Sree Veerabhadrakavu, Klayikkod (12°14'34.2"N; 75°9'55.2"E), Kasaragod district, Kerala, India, 19 m a.s.l., sacred grove, 21 October 2012, T. Augustine, M. Ramesan, S.P. Narayanan and S. Sathrumithra coll.; 11 clitellate, 12 aclitellate (ACESSD/EW/181), Sree Mannampurathukavu, Nileshwar (12°15'31"N; 75°7'58.1"E), Kasaragod district, Kerala, India, 13 m a.s.l., evergreen sacred grove, 20 October 2012, T. Augustine, S.P. Narayanan, S. Sathrumithra and M. Ramesan coll.; 8 clitellate, 13 aclitellate (ACESSD/EW/182), Kulangattumala Temple, Kadamgod (12°12'16.9"N; 75°8'3.9"E), Kasaragod district, Kerala, India, 7 m a.s.l., evergreen patch with litter, 21 October 2012, S.P. Narayanan, S. Sathrumithra, T. Augustine and M. Ramesan coll.

##### Diagnosis.

Length 60–70 mm, diameter 3.5 mm, 150–173 segments. Setae aa = 4.75–5.75 ab = 0.95–1.04 bc = 5.42–5.75 cd = 0.23–0.27 dd at segment 8; aa = 7.33–9 ab = 1–1.04 bc = 7.33–9 cd = 0.25–0.35 dd at segment 20. Male pores paired, at 10/11, slightly lateral to setae b lines. Spermathecal pores paired, at 7/8, at setae c lines. Genital markings paired, lateral to setae ab on segment 11, occasionally extending on to segment 12. Four gizzards in segments 13–16 or 14–17. Prostates glandular, hemi-ovoidal, sessile; vas deferens short, joining prostate at anterior and inner side. Spermathecae paired in segment 8; atrium cushion like ectal widening of spermathecal duct, partly embedded in body wall in segment 8, several times as wide as spermathecal duct.

##### Distribution.

India: Kerala: District Kasaragod: Adakasthala, Kadamgod, Klayikkod, Kottencheri, Nileshwar, Pandigaya, Periyathaduka, Plachikkara, Ranipuram and Veeramalakunnu (all new records) (Fig. 2); Karnataka: District Uttara Kannada (North Kanara): Talewadi, Castle Rock (Stephenson 1917); District Shivamogga (Shimoga): Batkal, Kogar, Nakkalu (Blanchart and Julka 1997).

##### Remarks.

Range of the length, diameter, and number of segments of the Kerala specimens are 38–51 mm, 3 mm, and 166–177 respectively. Gizzards are in segments 14–17.

#### Drawida modesta

Taxon classificationAnimaliaOligochaetaMoniligastridae

Rao, 1921

Drawida modesta Rao, 1921. Ann. Mag. Nat. Hist. (ser. 9), 8: 525.
Drawida modesta , Stephenson 1923. Fauna Br. India, Oligochaeta: 145.

##### Material examined.

14 clitellate, 4 aclitellate (ACESSD/EW/160), Chandanathodu - Kannavam range (11°51'3.2"N; 75°48'12"E), Kannur district, Kerala, India, 784 m a.s.l., evergreen forest, 01 December 2012, S.P. Narayanan, T. Augustine and S. Sathrumithra coll.; 3 clitellate (ACESSD/EW/166), Chandanathodu - Periya Range (11°50'44.6"N; 75°48'27.4"E), Wayanad district, Kerala, India, 778 m a.s.l., evergreen forest, 01 December 2012, T. Augustine, S. Sathrumithra and S.P. Narayanan coll.; 1 clitellate, 6 aclitellate (ACESSD/EW/399), Perumalkunnu (11°53'50.1"N; 75°54'16.3"E), Kannur district, Kerala, India, 1076 m a.s.l., grassland and evergreen forest, 02 December 2012, S.P. Narayanan, T. Augustine and S. Sathrumithra coll.; 14 clitellate (ACESSD/EW/401), Kunnathoorpadi (12°4'55"N; 75°37'39.1"E), Kannur district, Kerala, India, 579 m a.s.l., evergreen forest with reed breaks, 30 November 2012, S.P. Narayanan, T. Augustine and S. Sathrumithra coll.; 1 clitellate (ACESSD/EW/402), Chandanathodu - Kottiyoor range (11°51'4.2"N; 75°47'12"E), Kannur district, Kerala, India, 714 m a.s.l., evergreen forest, 1 December 2012, S.P. Narayanan and T. Augustine coll.; 4 clitellate (ACESSD/EW/403), Nedumpoil (11°50'30.5"N; 75°46'39.1"E), Kannur district, Kerala, India, 365 m a.s.l., semi-evergreen forest, 1 December 2012, S.P. Narayanan, S. Sathrumithra and T. Augustine coll.; 7 clitellate, 2 aclitellate (ACESSD/EW/406), Koovattumoola – Thirunelli (11°54'3.8"N; 76°0'48.4"E), Wayanad district, Kerala, India, 782 m a.s.l., abandoned paddy field, 21 December 2012, A. Sasi, S.P. Narayanan and S. Sathrumithra coll.; 1 clitellate (ACESSD/EW/407), Paithalmala (12°10'1.7"N; 75°33'31.1"E), Kannur district, Kerala, India, 1076 m a.s.l., higher altitude evergreen forest, 30 November 2012, S.P. Narayanan, T. Augustine and S. Sathrumithra coll.; 5 clitellate (ACESSD/EW/409), Ambayithodu (11°51'50.8"N; 75°53'37.2"E), Kannur district, Kerala, India, 216 m a.s.l., disturbed mixed forest area, 02 December 2012, S.P. Narayanan, T. Augustine and S. Sathrumithra coll.; 14 clitellate, 8 aclitellate (ACESSD/EW/472), below Kakkadampoyil (11°19'41.9"N; 76°9'54.8"E), Malappuram district, Kerala, India, 98 m a.s.l., semi-evergreen forest near to a stream, 29 October 2014, S.P. Narayanan and S. Sathrumithra coll.; 1 clitellate (ACESSD/EW/473), Naadukaani (11°25'58"N; 76°23' 18.7"E), Malappuram district, Kerala, India, 530 m a.s.l., evergreen forest, 28 October 2014, S.P. Narayanan and S. Sathrumithra coll.

##### Diagnosis.

Length 75 mm, diameter 4 mm, 207 segments. Intersegmental furrow 1/2 very faint. Setae aa = 13.3 ab = 0.7 bc = 13.3 cd on segment 7, aa = 15.3 ab = 0.7 bc = 15.3 cd on segment 35. Male pores paired, transverse slits with prominent lips, at 10/11, slightly lateral to setae b lines. Spermathecal pores paired, at 7/8, at or slightly lateral to setae b lines. Genital markings paired, oval shaped on segment 7, just anterior to spermathecal pores. Gizzards 2, in segments 13–14. Prostates paired, glandular, sessile, circular to oval in shape; vas deferens short, discharging at dorsal surface and centre of prostates. Spermathecae paired in segment 8; atrium absent.

##### Distribution.

India: Kerala: District Kannur: Ambayithodu, Chandanathodu - Kannavam range, Chandanathodu - Kottiyoor range, Kunnathoorpadi, Nedumpoil, Paithalmala, Perumalkunnu; District Wayanad: Chandanathodu - Kannavam range, Koovattumoola – Thirunelli; District Malappuram: Kakkadampoyil, Naadukaani (all new records) (Fig. 2); Karnataka: District Kodagu (Coorg): Moornad, Coorg Hills (Rao 1921).

##### Remarks.

Range of the length, diameter, and number of segments of the Kerala specimens are 60–72 mm, 3–4 mm, and 151–197 respectively. Genital markings are absent in some specimens from Kerala (Table 3).

**Table 3.** Number of *Drawida modesta* specimens with and without genital markings (GM).

Site & Reg. no.	With GM	Without GM	Total
Chandanathodu (Periya range); ACESSD/EW/166	1	2	3
Perumalkunnu; ACESSD/EW/399	3	4	7
Kunnathoorpadi; ACESSD/EW/401	4	10	14
Nedumpoil; ACESSD/EW/403	3	1	4
Ambayithodu; ACESSD/EW/409	4	1	5
Paithalmala; ACESSD/EW/407	1	0	1
Koovathumoola; ACESSD/EW/406	8	1	9
Chandanathodu (Kottiyoor range); ACESSD/EW/402	0	1	1
Total	22	20	42

#### Drawida somavarpatana

Taxon classificationAnimaliaOligochaetaMoniligastridae

Rao, 1921

Drawida somavarpatana Rao, 1921. Ann. Mag. Nat. Hist. (ser. 9), 8: 497.
Drawida somavarpatana , Stephenson 1923. Fauna Br. India, Oligochaeta: 158.

##### Material examined.

6 clitellate (ACESSD/EW/405), Ranipuram (12°25'5.7"N; 75°21'4.4"E), Kasaragod district, India, 935 m a.s.l., grassland and evergreen forest, 17 December 2013, S.P. Narayanan and S. Sathrumithra coll.; 2 clitellate (ACESSD/EW/408), Paithalmala (12°10'1.7"N; 75°33'31.1"E), Kannur Dist., India, 1076 m a.s.l., higher altitude evergreen forest, 30 November 2012, S.P. Narayanan, T. Augustine and S. Sathrumithra coll.; 1 clitellate, 3 aclitellate (ACESSD/EW/410), Koombanmala (12°20'43.2"N; 75°24'41.4"E), Kasaragod district, Kerala, India, 867 m a.s.l., grassland, 20 October 2012, S.P. Narayanan, S. Sathrumithra, M. Ramesan and T. Augustine coll.; 1 clitellate, 1 aclitellate (ACESSD/EW/411), Kottathalachimala (12°15'53.1"N; 75°25'45"E), Kannur district, Kerala, India, 664 m a.s.l., deciduous forest, 28 November 2012, S. Sathrumithra, T. Augustine and S.P. Narayanan coll.; 2 clitellate (ACESSD/EW/412), Kunnathoorpadi (12°4'55"N; 75°37'39.1"E), Kannur district, Kerala, India, 579 m a.s.l., evergreen forest with reed breaks, 30 November 2012, S.P. Narayanan, T. Augustine and S. Sathrumithra coll.; 2 clitellate (ACESSD/EW/413), Sree Deviyottukavu sacred grove - Aalapadamba (12°12'34.1"N; 75°15'9.7"E), Kannur district, Kerala, India, 8 m a.s.l., evergreen sacred grove, 29 November 2012, S.P. Narayanan, T. Augustine and S. Sathrumithra coll.; 9 clitellate, 12 aclitellate (ACESSD/EW/414), Kottencheri (12°20'57.6"N; 75°24'5.9"E), Kasaragod district, Kerala, India, 801 m a.s.l., evergreen forest, 20 October 2012, S.P. Narayanan, T. Augustine, S. Sathrumithra and M. Ramesan coll.; 4 aclitellate (ACESSD/EW/449), Panathoor (12°27'31.3"N; 75°20'42.9"E), Kasaragod district, Kerala, India, 98 m a.s.l., 24 October 2014, areca nut and coconut plantation, S. Sathrumithra coll.

##### Diagnosis.

Length 82–133 mm, diameter 4–6 mm, 111–154 segments. Ventral setae enlarged on pre-clitellar segments; aa = 5 ab = bc = 5 cd on segment 7, aa = 11 ab = 0.7 bc = 11 cd on segment 35. Male pores paired puckered orifices with tumid lips, slightly lateral to setae b lines, at 10/11. Spermathecal pores paired, small, at 7/8, at setae c lines or in bc but closer to c. Gizzards 3–5, in segments 15–20. Prostates glandular, bilobed; vas deferens discharges at junction of prostatic lobes. Spermathecae paired in segment 8; atrium bilobed, elongate and erect; one lobe in segment 7 and the other in segment 8; spermathecal duct discharges at junction of atrial lobes.

##### Distribution.

India: Kerala: District Kasaragod: Koombanmala, Kottencheri, Panathoor, Ranipuram; District Kannur: Kottathalachimala, Kunnathoorpadi, Paithalmala, Aalapadamba (all new records from Kerala) (Fig. 2); Karnataka: District Kodagu (Coorg): Somavarpatana, spelt as ‘Somvarpet’ (Stephenson 1923), Coorg Hills (Rao 1921).

##### Biology.

Spermathecae and male genitalia are absent in a number of worms from Kerala, indicating parthenogenetic mode of reproduction.

##### Remarks.

The diagnosis is based on the present material from Kerala, description as given by Stephenson (1923) and re-examination of type material in Zoological Survey of India, Kolkata (Reg. no. W416/1). Worms from Kerala are longer and stouter, and have a greater number of gizzards than worms from Karnataka (Rao 1921). Body dimensions and number of gizzards in Kerala worms are: average length 94.61 mm (range = 82–133 mm; n =13); average width 5.38 mm (range 5–6 mm; n = 17); average number of segments 128.23 (range 111–154; n = 13), gizzards 3–5, mainly located between segments 15–20 (Table 4).

**Table 4.** Length, width, number of segments, and number of gizzards in selected specimens of *Drawida somavarpatana* from various localities.

Site name & registration number	Length (mm)	Width (mm)	Number of segments	Number of gizzards (segments)
Koombanmala; ACESSD/EW/410	87	5	125	5 (16–20)
	83	5	119	5 (16–20)
	83	6	130	NC
Kottathalachimala; ACESSD/EW/411	82	5	104	5 (16–20)
	NM	6	113	NC
Kunnathoorpadi; ACESSD/EW/412	107	6	135	NC
	86	5	134	3 (17–19)
Sree Deviyottukavu – Alapadamba; ACESSD/EW/413	NM	5	NC	3 (18–20)
	NM	5	NC	3 (17–19)
Kottencheri; ACESSD/EW/414	133	6	154	4 (16–19)
	101	5	142	5 (15–19)
	97	5.5	144	4 (16–19)
Ranipuram; ACESSD/EW/405	91	5.5	131	3 (18–20)
	95	5.5	125	4 (17–20)
	NM	5	NC	4 (16–19)
	89	6	111	4 (16–19)
	96	5	NC	4 (15–19)
	NM	NM	NC	5 (15–19)

NM – not measured, NC – not counted.

#### Drawida thurstoni

Taxon classificationAnimaliaOligochaetaMoniligastridae

Gates, 1945

Drawida thurstoni Gates, 1945. Jl. R. Asiat. Soc. Bengal, 11: 71.

##### Material examined.

2 clitellate, 4 aclitellate (ACESSD/EW/183), Kanjipara (11°26'16.4"N; 76°7'41.7"E), Kozhikode district, Kerala, India, 2000 m a.s.l., Shola forest, 23 December 2012, T. Augustine, S.P. Narayanan, A. Sasi and S. Sathrumithra coll.

##### Diagnosis.

Length 185–220 mm, diameter 7–8 mm, 166–187 segments. Setae aa = 12–13.33 ab = 1.06–1.11 bc = 12–16 cd = 0.21–0.23 dd at segment 8; aa = 13.66–14 ab = 1.13–1.23 bc = 20.5–21 cd = 0.24–0.28 dd at segment 20. Male pores paired, transverse slits at 10/11, about mid bc. Spermathecal pores paired, minute at 7/8, close to setae c lines. Nephridiopores on or close to setae d lines, occasionally dislocated into setae a, b on some segments behind clitellum. Genital markings absent. Gizzards 5, in segments 14–22. Prostates glandular, mushroom-shaped, stalked and erect, laterally compressed, glandular lining restricted to ental end; prostatic capsule vertical, ovoid; vas deferens coiled in to a cluster of hairpin loops; vas discharging into anterior face of prostate at ectal end. Spermathecae paired in segment 8; atrium irregularly ovoid, covered over by a thin layer of muscle, in segment 7 only or slightly extending to segment 8.

##### Distribution.

India: Kerala: District Kozhikode: Kanjipara (new record) (Fig. 2); Tamil Nadu: District Nilgiris: Nilgiri Hills (Gates 1945).

##### Remarks.

Range of the length, diameter, and number of segments of the Kerala specimens are 171–176 mm, 7.5–8 mm, and 183–185 respectively.

### Discussion

Earthworms of the primitive family Moniligastridae are believed to have originated somewhere in the region of Malaya Archipelago (Gates 1972, Blakemore et al. 2014); Jamieson (1977) suggests their origin near Myanmar. Its natural range encompass, south, southeast and east Asia, from peninsular India to Japan through Myanmar, China, extreme southern portion of Far East Russia, Korea, the Philippines, Borneo, and Sumatra (Gates 1972). The large range is primarily due to the spread of *Drawida*, the most speciose moniligastrid genus that presumably colonized peninsular India after the collision of Indian plate with Asia during Caenozoic period (Gates 1972, Blakemore et al. 2014). Recent attempts to resolve conflicts within the taxonomy of the genus have used molecular mtDNA barcoding, where possible of primary types (Blakemore et al. 2010, 2014; Blakemore and Kupriyanova 2010).

*Drawida* has undergone extensive radiation in India with 72 species listed by Blakemore (2007) but its greatest concentration (43 species) is found in the Western Ghats (Stephenson 1923, Gates 1945). Within the Western Ghats, the genus has an important centre of speciation in the southernmost state of Kerala, most species being endemic; there are 16 species, ten of which are endemic (Narayanan et al. 2016). The present discovery of two new species and five new records of *Drawida* further contribute to the vast species richness of the genus in the state.

## Supplementary Material

XML Treatment for Drawida

XML Treatment for Drawida polydiverticulata

XML Treatment for Drawida thomasi

XML Treatment for Drawida elegans

XML Treatment for Drawida kanarensis

XML Treatment for Drawida modesta

XML Treatment for Drawida somavarpatana

XML Treatment for Drawida thurstoni
